# Mo_3_Ni_2_N Nanoparticle Generation by Spark Discharge

**DOI:** 10.3390/ma16031113

**Published:** 2023-01-27

**Authors:** Jonas Elmroth Nordlander, Marie Bermeo, Pau Ternero, David Wahlqvist, Toni Schmeida, Sara Blomberg, Maria E. Messing, Martin Ek, Julia-Maria Hübner

**Affiliations:** 1 Department of Chemical Engineering and NanoLund, Lund University, P.O. Box 124, 22100 Lund, Sweden; 2 Department of Physics and NanoLund, Lund University, P.O. Box 118, 22100 Lund, Sweden; 3 Department of Chemistry and NanoLund, Lund University, P.O. Box 124, 22100 Lund, Sweden; 4 Leibniz-Institut für Festkörper- und Werkstoffforschung, Helmholtzstraße 20, 01069 Dresden, Germany

**Keywords:** metal nitrides, nanoparticles, spark discharge, carrier gas

## Abstract

Spark ablation is an advantageous method for the generation of metallic nanoparticles with defined particle sizes and compositions. The reaction of the metal particles with the carrier gas during the synthesis and, therefore, the incorporation of those light elements into structural voids or even compound formation was confirmed for hydrides and oxides but has only been suspected to occur for nitrides. In this study, dispersed nanoparticles of Mo_3_Ni_2_N and Mo with Janus morphology, and defined particle sizes were obtained by spark discharge generation as a result of carrier gas ionization and characterized using transmission electron microscopy and powder X-ray diffraction. Metal nitrides possess beneficial catalytic and thermoelectric properties, as well as high hardness and wear resistance. Therefore, this method offers the possibility of controlled synthesis of materials which are interesting for numerous applications.

## 1. Introduction

Metal nitrides are a class of ceramic materials possessing a number of beneficial properties, including excellent mechanical robustness due to their high hardness and wear resistance [1,2], high chemical stability, and electrical conductivity [3]. Their outstanding properties make metal nitrides suitable for a broad range of technological applications. For instance, BN is an important high-temperature ceramic material, whereas group 13 nitrides (AlN, GaN, InN) are used in opto- and microelectronic devices [4,5]. In thermoelectric materials, nitride inclusions can act as phonon scattering centers, resulting in reduced thermal conductivity and, therefore, in a higher figure of merit [6,7]. Furthermore, transition metal nitrides are of interest as cost-efficient alternatives compared to group VIII noble metals in diverse reactions, especially those involving heterogeneous catalysts [8,9,10], due to their similar electrocatalytic activity but without the downside of deactivation and poisoning [11]. Recently, the ternary nitride Mo_3_Ni_2_N was found to be an outstanding catalyst both for oxygen [12] and hydrogen evolution [13,14].

In general, the synthesis of metal nitrides can be achieved by a wide variety of strategies in accordance with the anticipated application, starting with classical methods including direct nitridation of the elements at high temperatures, thermal decomposition of metal amides, or the reduction of metal halides or oxides in the presence of nitrogen gas (for an extensive review of preparation techniques, see [15]). Nevertheless, heterogeneous catalysis requires high surface areas as one of the critical parameters determining the reaction rate [16]. The application of ammonolysis of different precursor compounds is one of the most widely used techniques to achieve a suitable morphology. However, heat management issues during synthesis [17], as well as various side products, poor crystallinity, or different particle sizes and shapes in the resulting products are often encountered. In contrast, spark discharge generation offers the possibility of size-selective synthesis of nanoparticles with defined particle concentrations [18]. With this method, Mo_3_Al_2_C-type (filled *β*-Mn structure) Mo_3_Ni_2_N nanoparticles were obtained. The nanoparticles were characterized using powder X-ray diffraction, transmission electron microscopy, and energy-dispersive X-ray spectroscopy.

## 2. Materials and Methods

### 2.1. Preparation

Nanoparticles were synthesized by spark discharge generation (for a detailed description of the method, see [18]). Ni or Ni_30_Mo_70_ rods (3 mm diameter) served as capacitor electrodes. The distance between the electrodes was kept constant, with a separation of 1.5 mm. N_2_ with 5% H_2_ was used as a carrier gas to avoid oxidation during particle formation [19]. Compaction of the particles was carried out at 1200 °C. The mobility diameter was set for size-selected particles to 30 nm with a differential mobility analyzer (DMA) located after the compaction furnace. For both size- and non-size-selected particles, the process was followed by deposition onto suitable substrates which were chosen according to the subsequent characterization method. Oxidization of the nanoparticles was performed in a muffle furnace at 400, 510, and 600 °C in air. For size-selected samples, a heating rate of 5 °C/min, followed by annealing for 5 min at the target temperature, and cooling with a rate of 15 °C/min were applied. For non-size-selected samples, a heating rate of 1 °C/min and a cooling rate of 15 °C/min were used.

### 2.2. Powder X-ray Diffraction (PXRD)

For PXRD samples, compacted particles were deposited on 4 × 4 or 5 × 5 mm SiO_x_ wafers (Siegert Wafer, J12004, lot no. 317559) without size selection to improve the deposition rate. The samples were transferred from the Si wafers to scotch tape, which was then loaded into the diffraction set-up. PXRD measurements were carried out in transmission mode using a STOE STADI MP X-ray diffractometer (Mythen 1k detector, Cu-*K*_α_ radiation, *λ* = 1.54178 Å).

### 2.3. Transmission Electron Microscopy (TEM)

Size-selected particle samples were created by directly depositing particles onto amorphous SiN grids (PELCO Prod. No. 21569CL-10, Batch CLEM-ULS-SN-MOS-A010(9)-T200-015_BL22) at a surface concentration of 90 μm^−2^. To characterize non-size selected samples (as-cast, see Table S1), particles from the SiO_x_ wafers as described for PXRD were transferred onto lacey carbon Cu grids by gently moving the grids across the sample surface. High-resolution imaging and energy-dispersive X-ray spectroscopy (EDX) mapping were carried out using a JEOL 3000F TEM (300 kV accelerating voltage) with an Oxford Instruments X-max 80 mm^2^ detector. EDX maps were acquired in scanning TEM (STEM) mode, using high-angle annular darkfield (HAADF) imaging to identify suitably oriented particles, and processed using routines from the HyperSpy [20] Python library. Curve-fitting was first carried out on the sum spectrum from the spectrum image to evaluate a fixed background model and characteristic X-ray peaks for each element (fixing energy and weightings). The spectrum image was then convoluted with a Gaussian filter (*σ* = 2 pixels) [21] before performing a linear fit of the previously determined peaks to the individual pixels. The corresponding maps, therefore, include contributions from all relevant peak families for each element. Standardless quantification of EDX spectra was conducted using the program INCA (Oxford Instruments). The simulation of EDX spectra was performed using the software DTSA-II [22,23]. Electron energy-loss spectroscopy (EELS) mappings were carried out using a probe-corrected FEI Titan TEM operated at 300 kV. The convergence and collection semi-angles were 22 and 56.5 mrad, respectively. The spectrometer dispersion was 0.5 eV, and the spectral range was 150–1175 eV.

## 3. Results and Discussion

**Figure 1:**
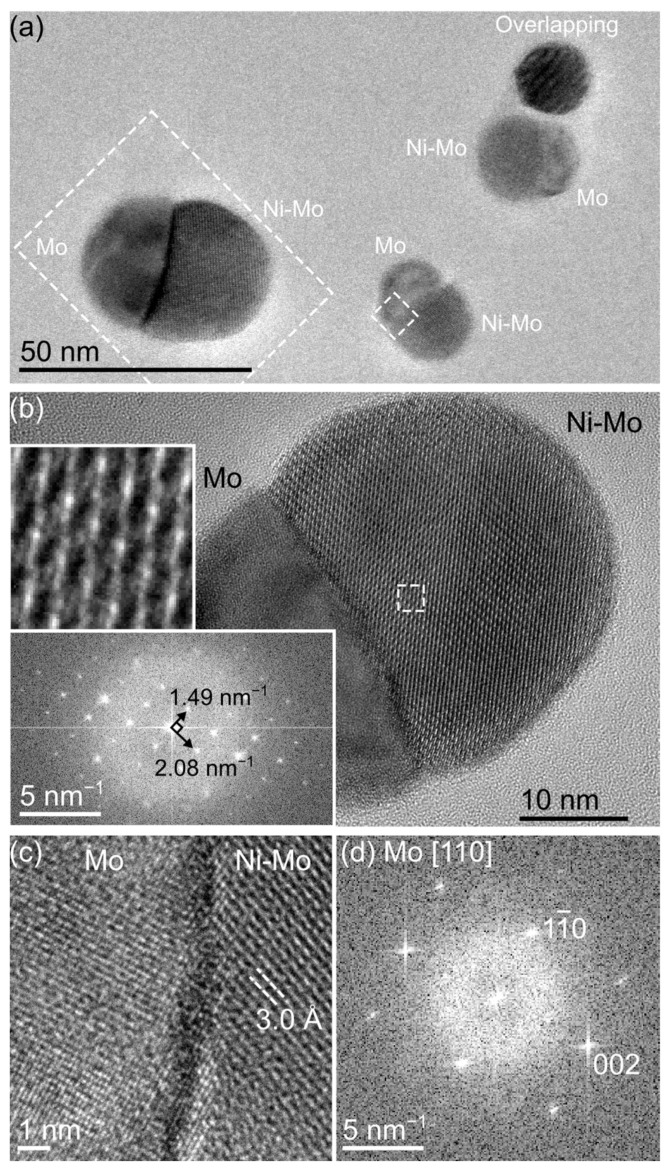
(**a**) Overview of an as-deposited Ni-Mo sample on an amorphous SiN substrate. The two phases are indicated for each particle. The marked regions are shown in more detail in subsequent panels. (**b**) HR-TEM image of the larger particle marked in (**a**), with insets showing the phase contrast pattern from a 2.5 × 2.5 nm^2^ region and corresponding diffractogram from the Ni-Mo phase (corresponding to the [001] zone of Mo_3_Ni_2_N, as described later). (**c**) HR-TEM image showing the interface between the two phases from the smaller particle marked in (**a**). The pure Mo phase shows lattice spacings corresponding to pure *bcc* Mo in a [110] viewing direction, as further shown by the diffractogram in (**d**) (excluding the Ni-Mo phase).

Bimetallic Ni-Mo nanoparticles with defined particle surface concentrations are synthesized by spark discharge generation (Table S1). By employing an integrated filtering system in the spark ablation system, particles with defined diameters of 30 nm are obtained (Figure 1a; note that a larger-than-usual particle is included in this overview image and is used for further analysis at higher resolution because it happened to be aligned close to the zone axis for the Ni-Mo phase).

**Figure 2:**
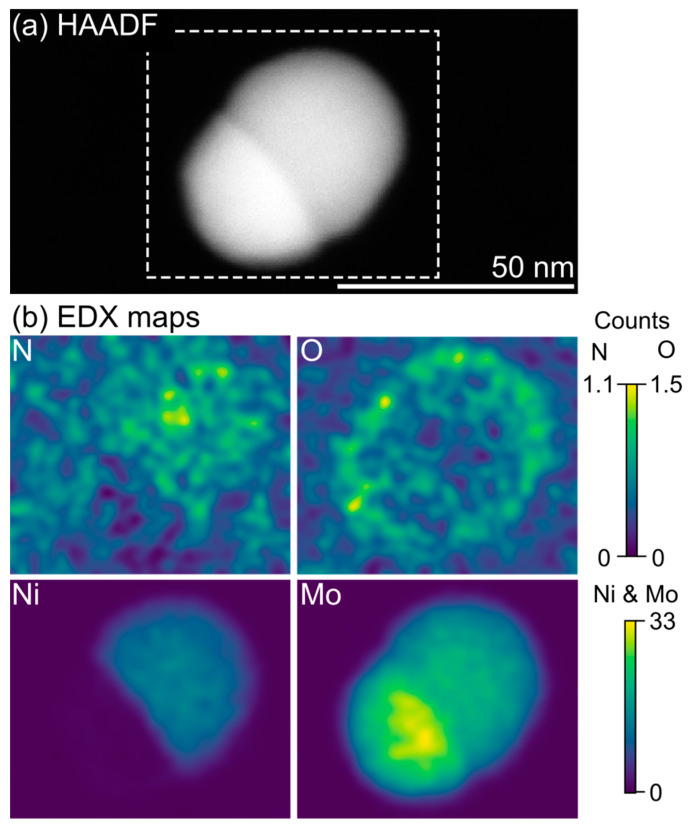
(**a**) STEM-HAADF overview image of a biphasic particle exhibiting a Janus morphology. (**b**) EDX mapping of the particle shows one phase containing elemental Mo (lower half) and the other containing an Ni-Mo compound with an increased N signal (upper half). Due to sample transfer being conducted in ambient air, the particles have a thin, oxidized shell.

As-deposited samples predominantly contain biphasic particles exhibiting a Janus morphology with two distinct halves (Figure 1b). The first phase can be easily identified based on HR-TEM images (Figure 1c,d) and STEM-EDX mapping (Figure 2) as elemental Mo. The second phase contains both Ni and Mo with a Ni:Mo ratio estimated by EDX to be 35:65. Furthermore, the EDX maps indicate that the bimetallic phase has incorporated N (Figure 2b). Simulated EDX spectra (Figure S1) and complementary EELS analysis (Figure S2) confirm the presence of N and the reliability of the EDX mapping. The level of N incorporation can, however, not be determined by either EDX (due to low signal from light elements) or EELS (due to overlap with Mo edges [24], which also prevents reliable mapping of the N distribution with this technique).

**Figure 3:**
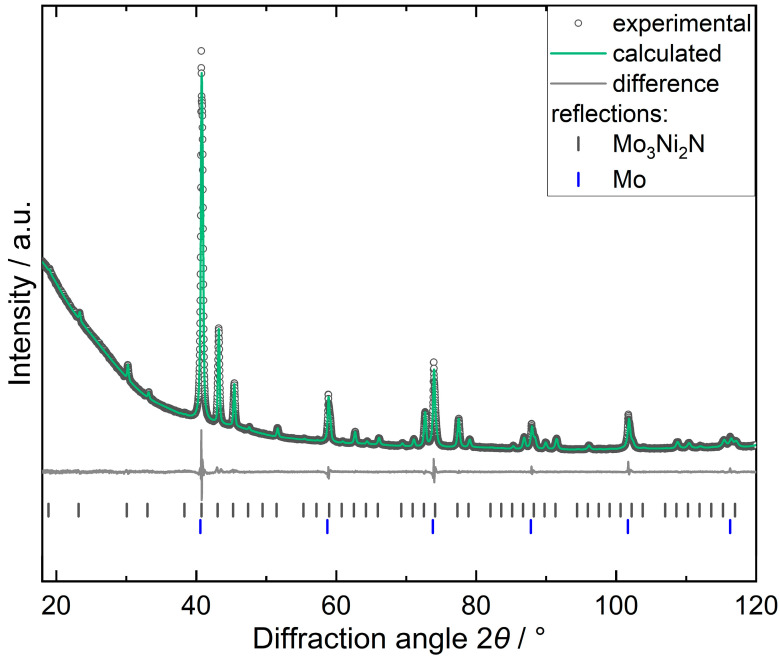
Rietveld refinement of Mo_3_Ni_2_N.

Identification and detailed characterization of the bimetallic phase require the application of PXRD (Figure 3). PXRD revealed Mo_3_Ni_2_N in addition to elemental Mo (Figure 3). Mo_3_Ni_2_N crystallizes in the cubic space group *P*4_1_32 and is isotypic to Mo_3_Al_2_C (filled *β*-Mn structure), with the lattice parameter *a* = 6.6394(1) Å. The unit cell dimensions are in accordance with high-resolution TEM images and selected area electron diffraction along the [001] zone axis (Figure 1b). The unit cell dimensions are in good agreement (difference of the lattice parameters <1%) with previous reports of Mo_3_Ni_2_N synthesized by ammonolysis [25] or by reduction–nitridation of NiMoO_4_ or mixed metal powders [26]. 

Rietveld refinement results in residuals *R* = 0.0169 and *wR* = 0.0198 (Table 1), which are lower than previous refinements comprising residuals *R* = 0.1107, *wR* = 0.1590 [25], and *wRp* = 0.0634 [26]. Therefore, the presented model provides an improved fit to the data. Both positional and displacement parameters were allowed to refine freely. All sites are fully occupied, and the displacement ellipsoids of Mo, Ni, and N are close to being spherical (Table 2).

**Figure 4:**
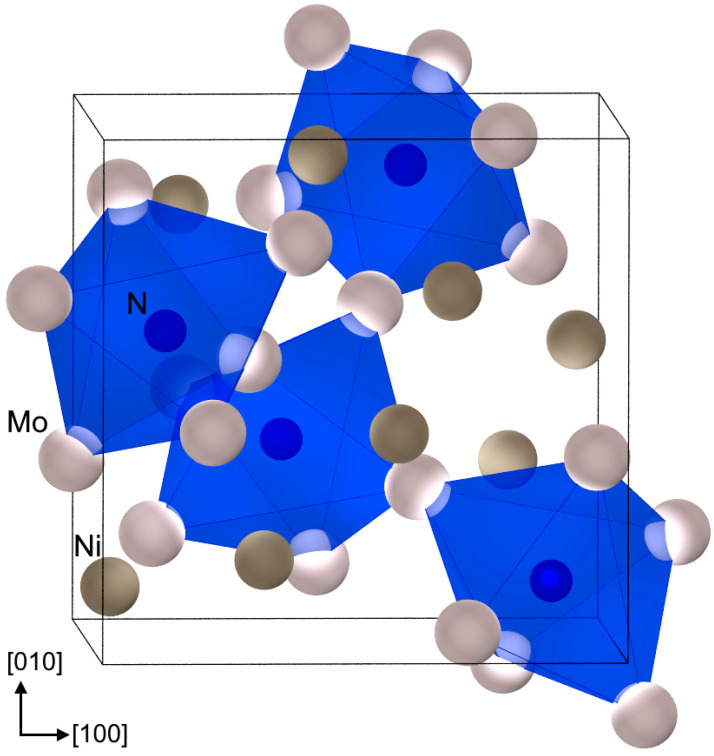
Crystal structure of Mo_3_Ni_2_N with corner-sharing distorted octahedra centered by N.

In Mo_3_Ni_2_N, N occupies the octahedral voids of a *β*-Mn type structure and is surrounded solely by Mo (Figure 4). The octahedral voids form a corner-sharing network. The coordination number of Mo is 14, and that of Ni is 12. The interatomic distances for Mo-N and Mo-Ni are in line with those of other Mo nitrides [27,28] and Mo-Ni compounds [29], respectively (Table 3). Interestingly, the homoatomic distances for Mo-Mo and Ni-Ni (Table 3) are very similar to the ones observed in the respective elements (deviation < 2%, [30,31]).

The overall Ni:Mo ratio in the biphasic nanoparticles is in good agreement with the initial electrode composition, as expected in the case of alloyed electrodes [32]. By PXRD, elemental Mo is observed and refined to a phase fraction of 47.5%. However, due to the very strong overlap of all observed Mo peaks with the primary phase, the accuracy of this result is expected to be very limited. EDX measurements instead provide more precise measurements from isolated particles (both in size-selected samples as well as for similar-size particles selected from non-size-selected samples). Here, an overall Mo fraction of 70% ± 5 at.-% Mo is observed (Table S2). These measurements are further complemented by analysis of the TEM images where the composition is estimated by assuming the two phases have spherical cap morphologies (Figure S3). In line with the EDX measurement and electrode composition, the image analysis yields a Mo fraction of 73% ± 2 at.-% (Table S3).

The Mo_3_Ni_2_N nanoparticles generated by spark discharge are stable and show good resilience toward oxidation. Storage in ambient air results only in a thin surface oxide layer, visible in the EDX maps (Figure 2b) but not identifiable as a separate crystalline surface phase in the HR-TEM images (Figure 1b). This surface layer appears self-limiting and does not increase measurably in thickness even after six months of storage (Figure S2). Heat treatment of the samples leads to the decomposition of the nitride compound and the formation of binary and ternary oxides (Table S1) in accordance with previous studies [25]. 

Whereas several examples of the formation of, e.g., hydrides and oxides by spark discharge generation [33,34,35,36,37] are known, the possibility of nitride formation as a result of carrier gas ionization during spark discharge generation was under discussion but not validated hitherto. In contrast, the methodologically related laser ablation synthesis was employed, e.g., to obtain aluminum nitride nanoparticles [38]. Spark discharge generation offers the opportunity for straightforward synthesis of metal nitride nanoparticles in addition to other established methods [39].

As mentioned above, the obtained nanoparticles show a Janus morphology with two distinct halves. The first half consists of Mo_3_Ni_2_N, which is of special interest as a heterogeneous catalyst [8,9,10] both for oxygen [12] and hydrogen evolution [13,14]. Molybdenum, present in the second half, was reported to act as an effective catalytic promoter either in the elemental form [40] or as oxide [41] or sulfide [42], potentially accessible after further sample treatment. The possibility of enhancement of the catalytic activity of Mo_3_Ni_2_N by the presence of Mo or further reaction products will be subject to future studies.

## 4. Conclusions

Nanoparticles with a Janus morphology of the nitride Mo_3_Ni_2_N and elemental Mo were obtained by spark discharge generation due to carrier gas ionization. The method offers the possibility of synthesizing well-dispersed nanoparticles with defined particle sizes. Therefore, further application to other technically relevant intermetallic systems has the potential for straightforward synthesis of high-quality nanocrystalline nitrides.

## Figures and Tables

**Table 1:** X-ray diffraction data for Mo_3_Ni_2_N. Further details on the crystal structure investigations can be obtained from the Fachinformationszentrum Karlsruhe, 76344 Eggenstein-Leopoldshafen, Germany (email: crysdata@fiz-karlsruhe.de, https://www.ccdc.cam.ac.uk/structures/? (accessed on 6 December 2022)) on quoting the depository numbers CSD-2218820.

Composition	Mo_3_Ni_2_N
Space group, Pearson symbol	*P*4_1_32 (No. 213), *cP*24
Unit cell parameters	
*a* [Å]	6.6394(1)
*V* [Å^3^]	292.67(1)
Formula units *Z*	4
Diffractometer	Stoe Stadi MP, Mythen 1k detector, Cu-Kα radiation, λ = 1.5406 Å, transmission mode
Measurement range, 2θ step	16.535 ≤ 2*θ* ≤ 121.085, *Δ*2*θ* = 0.015
Measured points/reflections	7740/46
Residuals and GOF	*R* = 0.0169, *wR* = 0.0198, GOF = 2.1451

**Table 2:** Position and displacement parameters for Mo_3_Ni_2_N.

Atom	Site	*a*/x	*b*/y	*c*/z	*U*_iso/aniso_
Mo	*12d*	1/8	0.2019(1)	0.4519(1)	0.0075(2)
Ni	*8c*	0.0675(1)	0.0675(1)	0.0675(1)	0.0045(2) *
N	*4a*	3/8	3/8	3/8	0.010(2)

* *U*_11_ = *U*_22_ = *U*_33_ = 0.0045(3); *U*_12_ = *U*_13_ = −0.0006(4); *U*_23_ = 0.0006(4).

**Table 3:** Selected interatomic distances in Mo_3_Ni_2_N.

Atoms		Distance/Å	Atoms		Distance/Å
Mo	2 N	2.0825(2)	Ni	3 Ni	2.469(1)
	2 Ni	2.7309(9)		3 Mo	2.7309(9)
	2 Ni	2.7459(9)		3 Mo	2.7459(9)
	4 Mo	2.7796(4)		3 Mo	2.8206(9)
	2 Mo	2.8153(5)			
	2 Ni	2.8206(9)	N	8 Mo	2.0825(2)

## Data Availability

CCDC 2218820 contains the supplementary crystallographic data for this paper. These data can be obtained free of charge via https://www.ccdc.cam.ac.uk/structures/? (accessed on 6 December 2022), or by emailing data_request@ccdc.cam.ac.uk, or by contacting The Cambridge Crystallographic Data Centre, 12 Union Road, Cambridge CB2 1EZ, UK; Fax: +44-1223-336033.

## Supplementary Materials

The following supporting information can be downloaded at: https://www.mdpi.com/article/10.3390/ma16031113/s1, Figure S1: STEM-EDX map; Figure S2: STEM-HAADF images and EEL spectra; Figure S3: Measurement scheme for particle volume calculation; Table S1: Synthesis conditions; Table S2: Particle composition; Table S3: Mo content in isolated particles. CCDC 2218820 contains the supplementary crystallographic data for this paper. These data can be obtained free of charge via https://www.ccdc.cam.ac.uk/structures/? (accessed on 6 December 2022), or by emailing data_request@ccdc.cam.ac.uk, or by contacting The Cambridge Crystallographic Data Centre, 12 Union Road, Cambridge CB2 1EZ, UK; Fax: +44-1223-336033.
