# Supplementary material for: Mo3Ni2N Nanoparticle Generation by Spark Discharge
Source: Materials (Basel). 2023 Jan 27;16(3):1113. doi: 10.3390/ma16031113 (PMC9920893; doi:10.3390/ma16031113)
Supplement: Supplementary file 1 [file materials-16-01113-s001.zip › materials-2116807-supplementary.pdf]

materials

SUPPORTING INFORMATION

Mo_3Ni_2N Nanoparticle Generation by Spark Discharge

Jonas Elmroth Nordlander [1], Marie Bermeo [2], Pau Ternero [2], David Wahlqvist [3], Toni Schmeida [4], Sara Blomberg [1], Maria E. Messing [2], Martin Ek [3] and Julia-Maria Hübner [3,*]

[1] Department of Chemical Engineering and NanoLund, Lund University, Box 124, 22100 Lund, Sweden
[2] Department of Physics and NanoLund, Lund University, Box 118, 22100 Lund, Sweden
[3] Department of Chemistry and NanoLund, Lund University, Box 124, 22100 Lund, Sweden
[4] Leibniz-Institut für Festkörper- und Werkstoffforschung, Helmholtzstraße 20, 01069 Dresden, Germany

Table of contents

1. Synthesis

2. Confirmation of N incorporation from simulated EDX spectra and experimental EELS

3. Quantifications of Mo fraction based on EDX and TEM image analysis

1. Synthesis

Table S1. Synthesis conditions of non-size selected samples and obtained compounds.

Sample number	Material	Target particle surface concentration / µm^{-2}	Heat treatment	Detected phases (PXRD)
1a	Ni electrodes, SiO_x wafers	7000	As-cast	Ni
1b			1 h at 600°C in air	NiO
1c			7h at 600°C in air	NiO
3a	$Ni_{30}Mo_{70}$ electrodes, SiO_x wafers	7000	1h at 600°C in air	α-$NiMoO_4$, β-$NiMoO_4$, MoO_3
3b			4h at 600°C in air	α-$NiMoO_4$, β-$NiMoO_4$, MoO_3
3c			7h at 600°C in air	α-$NiMoO_4$, β-$NiMoO_4$
3d			10h at 600°C in air	α-$NiMoO_4$, β-$NiMoO_4$
4a	$Ni_{30}Mo_{70}$ electrodes, SiO_x wafers	7000	As-cast	Mo_3Ni_2N, Mo
4b			As-cast	Mo_3Ni_2N, Mo
4c			As-cast	N/A
9a	$Ni_{30}Mo_{70}$ electrodes, SiO_x wafers	3500	400°C, 4h in air	α-$NiMoO_4$, β-$NiMoO_4$, MoO_3
9b			400°C, 4h in air	α-$NiMoO_4$, β-$NiMoO_4$, MoO_3
9c			510°C, 4h in air	α-$NiMoO_4$, MoO_3
9d			510°C, 4h in air	α-$NiMoO_4$, MoO_3

2. Confirmation of N incorporation from simulated EDX spectra and experimental EELS

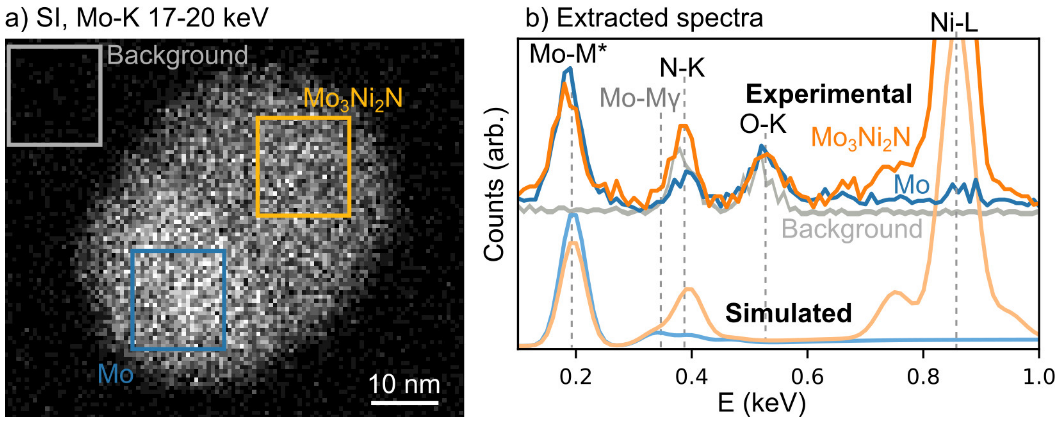

Figure S1. (a) STEM-EDX map (of the Mo-K signal) from which sum spectra of the two phases were extracted as indicated by the two marked regions. (b) The extracted experimental spectra are in good agreement with simulated spectra. The spectra were simulated using DTSA-II with the following parameters: 25 nm (Mo) or 30 nm (Mo_3Ni_2N) thick films over vacuum with densities of 10.2 and 9.5 g/cm3 respectively, 300 kV beam energy, silicon drift detector (0.032 sR collection angle and 128 eV energy resolution) with Moxtek AP 3.3 window, 60 s live-time, 1 nA beam current. The spectra were scaled manually based on the Mo-M* peak at around 0.2 keV. The simulations indicate that the majority of the signal at 0.4 keV stems from N (N-K) rather than Mo (Mo-Mγ). Note that in the experimental spectra, there is also a N-K contribution from the SiNx sample support. However, since the added signal from the sample support is constant, the N-K signal at 0.4 keV can be used for showing the relative distribution of N. However, the Mo-$M_{2,3}$ edge at 0.4 keV further complicates the quantitative interpretation of the N-K signal. The absorption effect is particularly noticeable in the Mo side of the particle, where the background signal from the underlying SiN is smaller than outside the nanoparticle, c.f. the background and Mo spectra in (b) (and also Figure 2 in the main text). Likewise, absorption of X-rays in the Mo_3Ni_2N phase explains the low N-K signal from this phase. The complex combination of additional N-K signal from the sample support and absorption of the same by Mo prevents quantification of the N content from EDX. Furthermore, these effects make it impossible to discount small amounts of N in the Mo phase. The signal at 0.5 keV seen exclusively in the experimental data stems from the surface oxide (O-K).

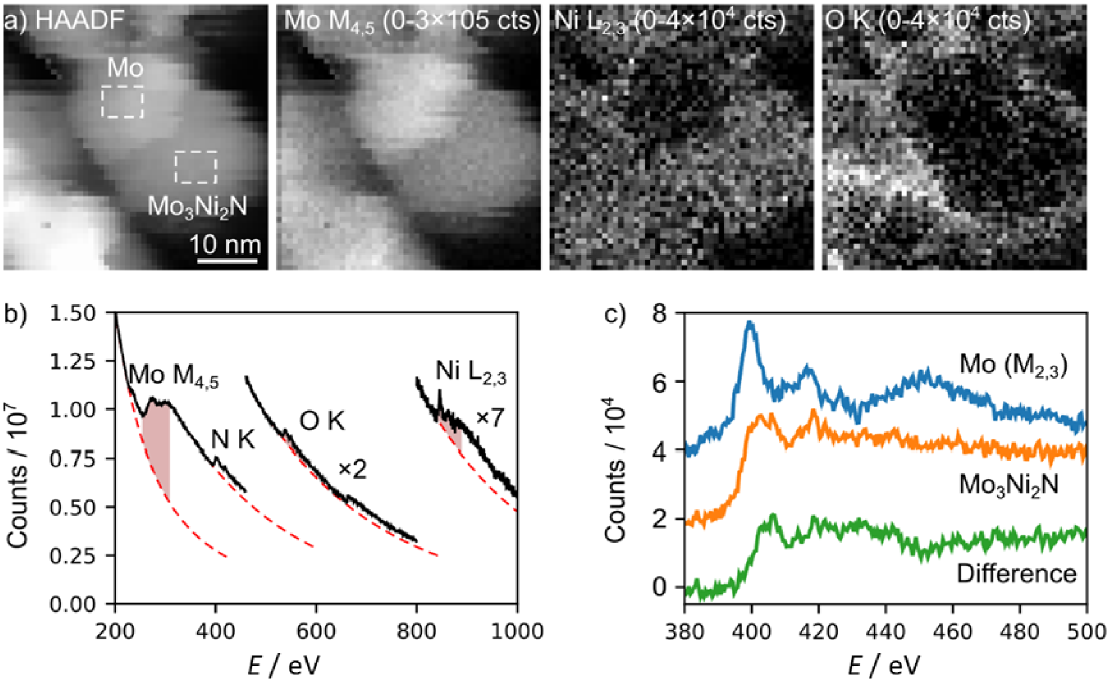

Figure S2. (a) STEM-HAADF image acquired simultaneously with EELS showing the distribution of Mo (Mo-$M_{4,5}$ 255-305 eV), Ni (Ni-$L_{2,3}$ 839-889 eV) and O (O-K 533-568 eV). (b) The sum spectrum from (a) is shown together with background models (1st order log-polynomials) and signal integration windows. (c) The N-K spectral region from the two phases show a strong contribution from Mo-$M_{2,3}$ for both the pure Mo and the bimetallic Ni-Mo phases. However, by subtracting the Mo-$M_{2,3}$ signal from the Ni-Mo spectrum, the presence of an excess signal related to N is revealed. Samples were stored in air for six months prior to STEM-HAADF analysis; the low oxygen content in spite of this storage demonstrates the particles' stability towards oxidation.

3. Quantifications of Mo fraction based on EDX and TEM image analysis

Table S2. Mo percentages from EDX measurements of Ni and Mo for isolated particles.

Particle no.	Mo / at.%	Ni / at.%
1	70.30	29.70
2	69.99	30.01
3	66.48	33.52
4	73.73	26.27
5	74.06	25.94
6	67.93	32.07
7	65.22	34.78
8	79,96	20.04
9	61.40	38.60
10	64.28	35.72
11	74.85	25.15
**Average**	**69.84**	**30.16**
**Std. Dev.**	**5.21**	**5.21**

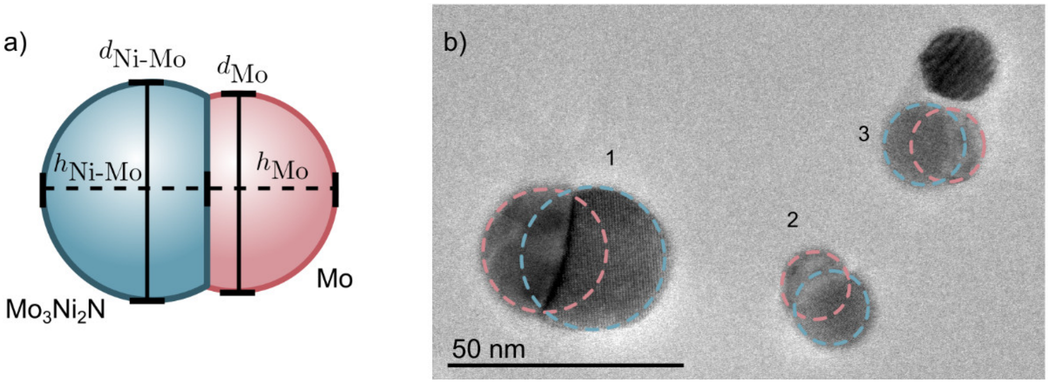

Figure S3. (a) Schematic of measurement extracted from (b) TEM images to calculate the volume of the two phases (*bcc* Mo labeled "Mo" and Mo_3Ni_2N "Ni-Mo") as spherical caps.

Table S3. Summary of measurement from 10 particles based on the model illustrated in Figure S3. Unit cell parameters of 0.315 nm (containing 2×Mo) and 0.664 nm (containing 4×Mo_3Ni_2N) were used to calculate the number of atoms, and subsequently the Mo fraction.

Particle	$d_{\text{Ni-Mo}}$ / nm	$h_{\text{Ni-Mo}}$ / nm	d_{Mo} / nm	h_{Mo} /nm	Mo / at.%
1	30.9	23.1	27.6	17.5	73
2	17.5	12.9	15.8	9	72
3	16.7	12.8	15.3	8.8	72
4	20.4	12.4	15.9	9.1	71
5	19.1	13.8	17.5	9.1	72
6	19.5	11.8	16.6	11.1	76
7	19.2	12.8	17.3	9.1	72
8	19	13.1	17.5	10.5	75
9	14.3	9.3	13.3	8.4	77
10	20.4	12.4	18.9	8.5	72
**Average**					**73**
**Std. Dev.**					**2**
